# Influence of information attributes on information dissemination in public health emergencies

**DOI:** 10.1057/s41599-022-01278-2

**Published:** 2022-08-05

**Authors:** Meng Cai, Han Luo, Xiao Meng, Ying Cui, Wei Wang

**Affiliations:** 1 School of Humanities and Social Sciences, Xi’an Jiaotong University, Xi’an, China; 2 School of Journalism and New Media, Xi’an Jiaotong University, Xi’an, China; 3 School of Mechano-Electronic Engineering, Xidian University, Xi’an, China; 4 School of Public Health, Chongqing Medical University, Chongqing, China

**Keywords:** Complex networks, Health humanities

## Abstract

When public health emergencies occur, relevant information containing different topics, sentiments, and emotions spread rapidly on social media. From the cognitive and emotional dimensions, this paper explores the relationship between information attributes and information dissemination behavior. At the same time, the moderating role of the media factor (user influence) and the time factor (life cycle) in information attributes and information transmission is also discussed. The results confirm differences in the spread of posts under different topic types, sentiment types, and emotion types on social media. At the same time, the study also found that posts published by users with a high number of followers and users of a media type are more likely to spread on social media. In addition, the study also found that posts with different information attributes are easier to spread on social media during the outbreak and recurrence periods. The driving effect of life cycles is more obvious, especially for topics of prayer and fact, negative sentiment, emotions of fear, and anger. Relevant findings have specific contributions to the information governance of public opinion, the development of social media theory, and the maintenance of network order, which can further weaken the negative impact of information epidemic in the occurrence of public health emergencies, maintain normal social order, and thus create favorable conditions for the further promotion of global recovery.

## Introduction

With the development of information communication technology (ICT), the prospect of the digital age has been displayed today. Significant changes have taken place in the way and nature of information generation, circulation, and reception. New communication channels represented by social media such as Twitter, Facebook, and Weibo have gradually replaced traditional communication channels as the primary communication channels in people’s daily life. In the information age, we accept, utilize and disseminate all kinds of information at an alarming speed and quantity that subverts the past, and builds a substantial social network with the help of social media (De Paor and Heravi, 2020). In addition, thanks to advances in communications and networking technology, the form of one-way communication represented by newspapers, radio, television, and other media has been completely changed. With the help of social media platforms, new communication spaces have been established. Two-way or even multi-way communication provides more convenient communication channels for the public, which also means that the sender and receiver of the information can communicate anytime and anywhere across the distance between time and space (Capriotti and Ruesja, 2018; Han et al., 2015; Schneider and Check, 2010). Using this feature, social media has become an important communication channel for people to discuss events, share opinions, and interact.

In addition, with the convenience and immediacy of communication, social media has become an indispensable part of people’s daily life. According to statistical results, by 2020, the number of people using the Internet in the world has exceeded 4.5 billion, among which the number of social media users has exceeded 3.8 billion, and nearly 60% of users in the world are connected by digital information (Digital 2021: April Global Statshot, 2021). Users can easily interact with other users through specific topics and tags to discuss event-related content and information (Aladwani, 2015). At the same time, social media also becomes an essential platform for public information and emotions when a crisis occurs. With the help of social networks built by social media platforms, people in crisis events are no longer limited to seeking help through traditional ways as before. On the contrary, long before news organizations published relevant events, people had already used the power of social networks to obtain and publish crisis-related content on social media platforms such as Twitter. It also provides an important channel for understanding incidents’ relevant information and realizing emergency management (Martínez-Rojas et al., 2018). When emergencies occur, timely and accurate information sources are an important guarantee to rescue, save lives, reduce losses and improve the disaster relief process. Therefore, it is crucial to know relevant information about emergencies through social media (Brengarth and Mujkic, 2016).

As a software platform with a wide range of influences and instant messaging and sharing functions, the number of people using social media to collect health information to deal with public health emergencies increases yearly (Chu et al., 2017). Compared with traditional search engines, more effective social media platforms do not limit information acquisition to peer-to-peer communication but directly connect patients with people worried about diseases through the formation of various healthy communities, creating simple channels for the exchange and sharing of health information (Zhao and Zhang, 2017). Therefore, social media is still one of the most important choices for the public to obtain health information, although the lack of regulatory measures and anonymity mechanisms make false information, fake news, rumors and other misinformation on social media spread under the influence of various rumor strategies (Huang et al., 2020) (Zhao et al., 2021). For example, during the COVID-19 outbreak in December 2019, local governments took intervention measures to limit the spread of the virus, such as restricting the movement of people and increasing social distancing, given that the virus often spreads from person to person (He et al., 2022). As a result, social media has become the best choice for people to communicate, and it is also one of the main channels for updating and spreading information about the COVID-19 epidemic. The public can learn about the governance of public crises and related information from social media (Bao et al., 2020). In addition, COVID-19 has posed a serious challenge to the response of governments at different levels. It is particularly important to anticipate the need for physical and emotional resources to further reduce the negative impact of the virus (Vassallo et al., 2021). The rich data sources provided by social media help us to formulate more accurate governance solutions from the perspectives of social computing and data science.

When a public health emergency occurs, the public expresses their views and opinions on social media platforms. At the same time, users interact and communicate with other users through like, forwarding or comments, to build a social network related to the emergency (Chung and Zeng, 2020). However, corresponding to informative posts with simple forms and rich contents, the public under the influence of emergencies often ignores the form and conciseness of information but tends to post messages with an emotional tone, vague semantics, and a large number of exclamatory sentences on social media (Ghafarian and Yazdi, 2020). For policymakers and emergency managers, the posts about emergencies on social media also support them to understand public sentiment and formulate coping strategies, to realize better public opinion management (De Choudhury et al., 2014; Tettegah, 2016). At the same time, emotion also plays an important role in the spread of posts on social media. Under the influence of different emotions, posts related to emergencies also show great differences in the amount of forwarding (Yoon et al., 2016).

Looking back at the existing literature, a few scholars found that users of social media promote their behaviors to change through the perception of risks, thus realizing the preventive effect on the crisis under the influence of crisis events. However, the existing research focuses more on the cognitive dimension of users, such as information content and communication behavior, but tends to ignore the power of the emotional dimension (Yoo et al., 2016; Yoo et al., 2018). Even in the related research on emotional variables to explore users’ communication behavior, most of them take natural disasters and other events as the research objects and lack related research on public health emergencies (Li et al., 2020a, 2020b, 2020c, 2020d). Compared with natural disasters, accident disasters, and social security incidents, public health emergencies have a more lasting impact and a more comprehensive spatial range. The characteristics of long duration and fast diffusion also make public health events easy to evolve into global health crises and bring serious challenges to politics, economy, science, and other fields (McCloskey et al., 2020). Therefore, this study takes the COVID-19 epidemic, a public health emergency of large scale and long duration, as the research object, and brings the variables of cognitive dimension and emotional dimension into the research framework, trying to analyze the role of rational factors and perceptual factors on communication mechanism. It can not only make up for the deficiencies of existing researches on information transmission from the cognitive and sentiment dimensions, but also strengthen the understanding of the communication rules of public opinion caused by public health emergencies. Moreover, by comparing the differences in information transmission mechanisms between public health events and other emergencies, we can further weaken the negative impact brought by the information epidemic and create favorable conditions for global recovery.

In addition, from the perspective of the communication mechanism on social media, the communication process of posts on social media is also affected by other factors. Among them, the mechanism of media diversity is often neglected in the research. For example, due to different user types and influences, information published by different users varies greatly in the scope of influence and degree of diffusion on social media (Riquelme and González-Cantergiani, 2016). Posts published by users with high influence on social media are more likely to get attention from other users, and are more likely to be forwarded and disseminated by other users. (Hu et al., 2019). Besides media factors, time factors are also important factors affecting the communication mechanism (Xiao et al., 2020). The formation of public opinion on emergencies often goes through different life cycles. In different life cycles, users pay different attention to related events. At the same time, there are great differences in the propagation speed and diffusion quantity of event-related information on social media (Li et al., 2020a, 2020b, 2020c, 2020d). At the same time, with the change of life cycles, the development of emergencies may also change. During this period, there may be great differences between the content and emotion of information dissemination on social media (An et al., 2021). Although life cycles play a unique role in the communication mechanism of social media, the research on information dissemination often does not consider the important role of life cycles (Li et al., 2020a, 2020b, 2020c, 2020d). Or the research only studies from the perspective of the direct impact of life cycles on information transmission, ignoring the role of life cycles in the relationship between information attributes and information transmission (An et al., 2021). Therefore, it is necessary to consider the role of user and life cycles in the exploration of communication mechanisms, and further dig out the influence of conditional mechanisms on posts in the communication process.

Although existing researches have realized the interpretation of information transmission path from many aspects, they still face the following deficiencies. First, most existing studies have explained the conditions and paths of information transmission from the perspective of descriptive mechanisms, while causal analysis is relatively lacking (Shi et al., 2022). However, the identification and explanation of causal mechanisms will be more conducive to the in-depth excavation of the effective mechanism of information transmission (Teng et al., 2022). Second, most existing studies are based on a single dimension, such as the impact of information content and communication behavior on the mechanism of information transmission, while ignoring the effect of the sentiment dimension (Yoo et al., 2016; Yoo et al., 2018). It is difficult to explore the effective mechanism of information transmission more comprehensively. Thirdly, the dissemination of information in social networks is often not only directly affected by information attributes, but also by other indirect variables that can affect the dissemination of information (Li et al., 2020a, 2020b, 2020c, 2020d; Zhou et al., 2021). However, there are few studies on the influence of indirect variables on information transmission under the conditional path.

**Fig. 1:**
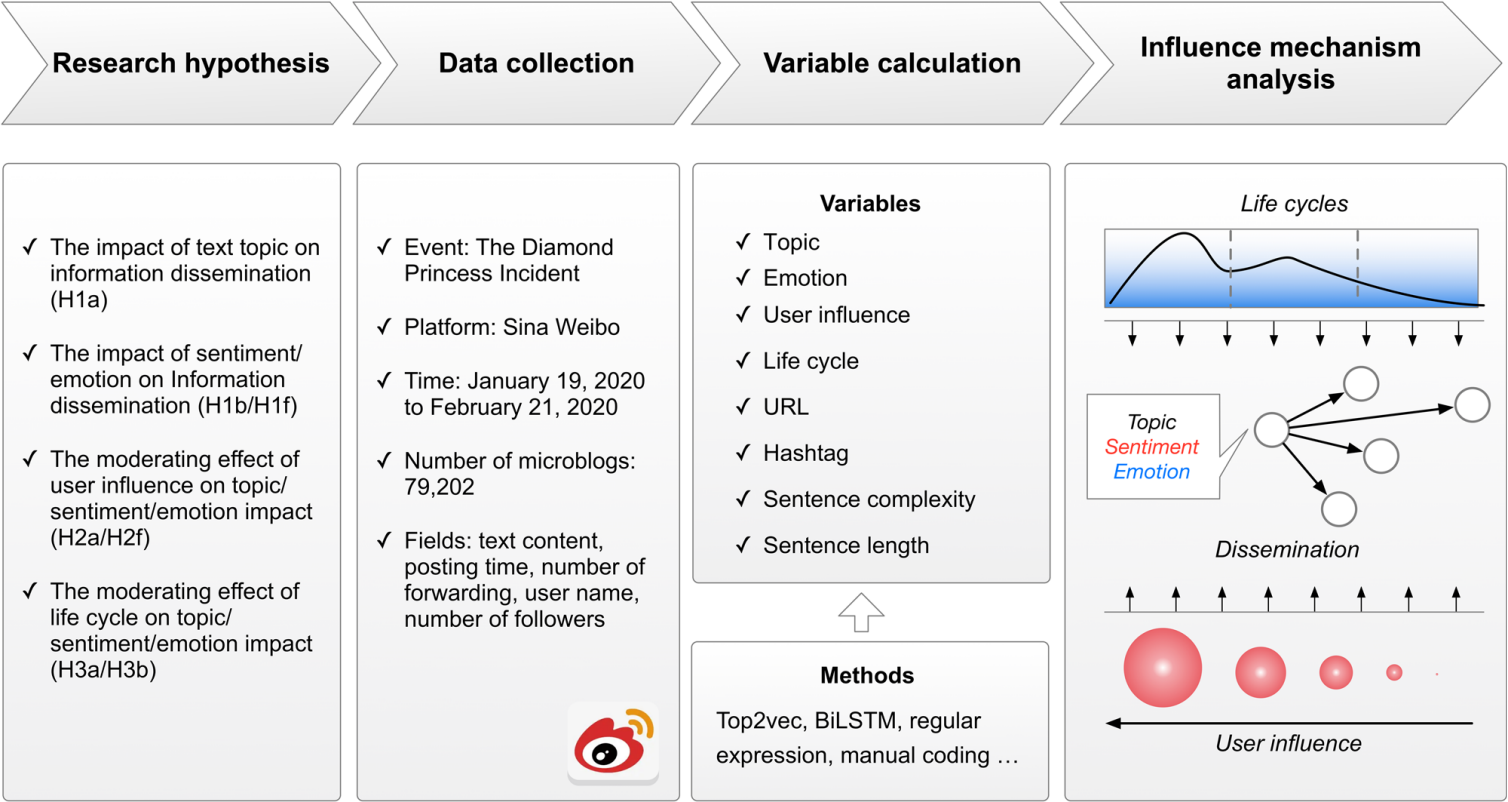
Research Framework. The main steps and flow of the research.

In order to bridge the research gap discussed above, this study aims to explore the influence mechanism of different information attributes on information dissemination in public health events. Specifically, to achieve this goal, this study established a hypothesis model of information diffusion according to planned behavior theory, sentiment contagion theory, influencer theory and other existing research findings. Then, data were collected from the Sina Weibo platform, which includes social media content and a variety of social media metadata. With the help of machine learning and deep learning methods, the concepts in the proposed hypothetical model were operated into concrete variables. Finally, the model proposed in this paper was statistically verified, which points out how the sentiment, emotion and topic attributes of information interact with the external environment such as the life cycles and the influence of publishers and influence the dissemination process. The research framework followed in this paper is shown in Fig. 1.

Therefore, compared with previous studies, the main contents and contributions of this study are as follows. Firstly, the study adopts a large-scale data set of social media, analyzes texts by deep learning method, and further explores the influence of text features by combining with regression model with strong explanatory power. It enriches the literature related to social media and communication behavior. Secondly, the cognitive dimension (text content) and sentiment dimension are considered to be included in the study to investigate their influence on communication behavior. Furthermore, sentiments are divided into different emotions, and the differences in users’ communication behaviors under different emotions are compared in detail. Thirdly, user factors and time factors are included in the analysis as moderating variables to further analyze the conditional mechanism of posts spreading on social media. It reveals the spreading path and influencing factors of posts more comprehensively. Therefore, the study not only further analyzed the public’s emergency response under the network public opinion, but also made corresponding contributions to the emergency management of public health emergencies.

The structure of other sections of this paper is as follows. In the second section, we propose our theoretical background and hypothesis based on the previous research. The third section puts forward the research methods of this paper, mainly involving data acquisition and processing and the specific design of the research. In the fourth section, we introduce the empirical analysis results and demonstrate the hypotheses put forward. Finally, in the fifth section, we put forward the research conclusion, explained the practical significance and theoretical contribution of the research, and put forward the further development direction and some research limitations of this paper.

## Literature review

### Text topics and information dissemination

In the study of individual behavior, planned behavior theory is one of the most commonly used theoretical frameworks, which is often quoted by scholars to explain and predict behaviors in many fields of human behavior (Sussman and Gifford, 2019; Yuriev et al., 2020). From the perspective of composition structure, the theory of planned behavior consists of three basic components, including behavioral attitude, subjective norm, and perceived behavioral control. Among them, the behavior attitude comes from the individual’s evaluation and view of the behavior. If the individual thinks that the implementation of the behavior will have a more positive result, the individual will hold a more positive attitude towards the behavior (Azjen, 1980). Subjective norm means that when an individual implements a certain behavior, the support or opposition of the majority of people around him exerts corresponding pressure on the individual, forcing the individual to adopt the opinions of the majority. Perceptual behavior control is an individual assessment of the feasibility of a specific behavior subconsciously (Ajzen, 1991). Based on the theory of planned behavior, individuals form certain behavioral tendencies under the combined action of behavioral attitude, subjective norms and perceived behavioral control. When an individual’s attitude towards a certain behavior is more intense, and the willingness of subjective norms and perceived behavior control is stronger, the possibility of implementing this behavior is stronger (Ajzen, 1985, 1991).

During the outbreak of COVID-19, social media became an important channel to establish communication between the government and the people, and between the people and the people. The harmfulness and long-term nature of COVID-19 also make the topics and emotions related to COVID-19 spread rapidly on social media, and have a continuous and important impact on public behavior during the epidemic (Han et al., 2020). Moreover, the behavior of individuals in the epidemic situation has a further influence on the epidemic dynamics, so that the epidemic situation usher in further changes (Wang et al., 2016). However, during the spread of topics, the influence and spread scope of different types of topic categories are not necessarily the same. When emergencies occur, there are often great differences in the dissemination of information under different topic categories on social media, among which topic in disaster response and recovery stages is more easily noticed and disseminated by the public (Imran and Castillo, 2015; Yu et al., 2019). Topics such as losses caused by disasters, how to deal with disasters, and heroes emerging in the rescue process can also arouse enthusiastic responses from lots of users (Wang et al., 2016). In the study of media information during the COVID-19 epidemic, as an important factor affecting the dissemination of information, the difference in topic categories also brings about the difference in the total amount of information on social media. Topics such as the economy and market under the epidemic, the spread and growth of cases, and suggestions for treatment and rehabilitation are more attractive to users than other topics, and spread more rapidly on social media (Chandrasekaran et al., 2020; Wahbeh et al., 2020).

According to the theory of planned behavior, the acquisition and dissemination of information under different topics on social media are mainly influenced by behavioral attitudes, subjective norms, and perceived behavioral control (Cameron, 2010; Yang and Wu, 2021). On the one hand, during the outbreak of COVID-19, information related to the epidemic spread widely and varied, and the public faced countless choices of information dissemination. Therefore, in the process of receiving and disseminating information, individual attitude is very important. Previous studies have linked the public’s attitude towards receiving information during the epidemic with risk perception. Under the effect of risk perception, epidemic prevention and prayer information are welcomed by the public (Adiyoso and Wilopo, 2021). On the other hand, due to the characteristics of fast information transmission, irrational emotions and group polarization on social media, the public is easy to be trapped by group users lacking audit mechanisms and spontaneously follow the crowd to spread their views of the majority under group pressure (Kwon et al., 2016). At the same time, before users obtain and forward information on specific content, they will also evaluate the information to be obtained to demonstrate whether the specific information can meet their own needs, thus achieving the preset goals (Hansen et al., 2018).

Previous studies have not yet reached an agreement on information dissemination under different topics. Based on the theory of planned behavior, this paper makes an empirical study on information dissemination during the COVID-19 epidemic. Considering that the health topic of praying to overcome diseases during the COVID-19 outbreak is more likely to be paid attention to by users, we put forward the following assumption (Adiyoso and Wilopo, 2021).

H1a: In public health emergencies, information about prayers is easier to spread on social media than information about other topics.

### Sentiment categories, emotion categories, and information dissemination

Compared with traditional communication platforms, social media occupies an advantageous position in both information dissemination and sentiment sharing (Lee and Sundar, 2013). At the same time, with the characteristics of low threshold, immediacy, and simple information release, social media has become one of the main channels of sentiment communication among the public. However, the large-scale discourse coordination and emotional resonance on social media platforms sometimes aggravate the imbalance of sentiments. The accumulation and resonance of negative emotions are more likely to cause large-scale group conflicts and cyber violence, which will cause serious challenges to the social order (Wei and Zhang, 2019). Therefore, taking public health emergencies as the research object and emotions as the influencing factors, it is particularly important to analyze the differences in the spread of different sentiments and emotions on social media.

Sentiment contagion theory constructed by social psychology has been recognized by scholars in explaining the relationship between individual emotional states and behavior. The theory holds that individual sentiment or emotional behavior may be formed under the influence of other people’s sentiments and emotions (Barsade, 2002; Hatfield et al., 1993). In recent years, the research has further expanded the application scope of sentiment contagion theory, and social networks are thought to have the power to promote emotional contagion on a large scale. Through the analysis of longitudinal data for 20 years, the study found that emotion has a transmission effect among people and has a long-term impact on other individuals (Fowler and Christakis, 2008). With the help of social media, the emotional state is transmitted to others under the action of emotion contagion, and it is enough for other users to feel the same emotion without knowing it (Kramer et al., 2014). At the same time, with the support of sentiment contagion theory, previous studies have also confirmed that sentiment is an important influencing factor in information dissemination. Under the action of different sentiments, information dissemination presents different trends and states (Kušen et al., 2017). For example, some studies have analyzed the relationship between sentiment and viral transmission of information and found that negative information can be transmitted more quickly and frequently than information containing positive or neutral sentiment (Tsugawa and Ohsaki, 2015). Similarly, negative sentiment can promote the dissemination of information more than positive sentiment, which has been proved in other studies (Steinert, 2020; Yeo et al., 2020).

However, at present, the influence of sentiment on information dissemination has not been unanimously recognized. Some studies have also found that information containing positive sentiment is far more likely to be disseminated than negative sentiment through empirical analysis (Ferrara and Yang, 2015a). Similar studies have found that positive sentiment can promote the dissemination of information, while negative sentiment can hinder the dissemination of information (Ferrara and Yang, 2015b; Kim et al., 2013). This is also a confirmation of the Pollyanna hypothesis, that positive sentiment is more popular than negative sentiment and neutral sentiment (Boucher and Osgood, 1969). During the COVID-19 epidemic, although the epidemic continued to bring heavy harm to people all over the world, it brought more symptoms such as depression, stress, and anxiety to the public (Wheaton et al., 2021). However, in predictable disasters, the popularity of positive posts is much higher than that of negative posts (Li et al., 2020a, 2020b, 2020c, 2020d). As a long-term disaster event, the response measures to the COVID-19 epidemic are more comprehensive and perfect, and the confidence in overcoming the epidemic is firmer. Based on this, this study puts forward the following hypotheses according to the sentiment contagion theory.

H1b: In public health emergencies, information containing positive sentiment is easier to spread on social media than information containing negative sentiment.

In addition, from the theoretical point of view of emotion evaluation, when individuals pay attention to important things that affect their happiness, emotions are formed in the evaluation of things (Moors et al., 2013). Based on the different things concerned, the emotions formed are also different (Roberts, 2003). There are also differences in the dissemination of information with different emotions on social media (Li et al., 2020a, 2020b, 2020c, 2020d). Reviewing the existing literature, some studies take the information dissemination of social security emergencies on Twitter as the research object and find that emotions such as anger and sadness are more likely to occupy a dominant position (Garcia and Rimé, 2019; Yeo et al., 2020). Some studies take public health emergencies as the research object and find that positive emotions such as gratitude and love can promote the dissemination of information on social media. Compared with sad or depressing information, most people prefer to forward information that contains positive emotions such as optimism and happiness that can make others feel happy (Jin et al., 2017; Lu et al., 2021). However, the role of different emotions on information dissemination has not yet reached an agreement, and the important influence of positive emotions and negative emotions on information dissemination is reflected in the research on the COVID-19 epidemic (Steinert, 2020; Zhao et al., 2020). Combining the previous literature and emotion-related theories, this study puts forward the following hypotheses.

H1c: In public health emergencies, information containing the emotion of like is easier to spread on social media.

H1d: In public health emergencies, information containing happiness is easier to spread on social media.

H1e: In public health emergencies, information containing fear is easier to spread on social media.

H1f: In public health emergencies, information containing sadness is easier to spread on social media.

H1g: In public health emergencies, information containing disgust is easier to spread on social media.

H1h: In public health emergencies, information containing anger is easier to spread on social media.

### Moderating effect of user influence

With the help of social media, users can interact with other users such as forwarding and commenting. However, in the process of information dissemination, the influence of users themselves is also an important factor affecting information dissemination. Conceptually, user influence is defined as the ability to influence the thoughts and behaviors of others (Cha et al., 2010). On the one hand, with the help of user influence, users can spread certain types of posts to their followers, increase the types of content they are interested in and promote the information dissemination of specific topic content (Zamparas et al., 2015). On the other hand, compared with users with low influence, users with higher influence can promote the spread of posts with different emotional types in information networks (Chung and Zeng, 2020). According to the theory of resource mobilization, as an important part of individual resources, the social network is a favorable tool for resource acquisition and influence expansion, and can provide important contributions to the realization of goals (McCarthy, 2005; Snow et al., 1980). The resource mobilization power of social networks is also reflected in the research on user influence. With social networks as a medium, a few influential users can have a significant impact on the entire network (Li et al., 2021). As one of the commonly used standard measurement units for measuring user influence, the number of users’ followers indicates the audience size and influence range of users, providing us with the most intuitive standard for measuring user influence. Based on the theory of resource mobilization, users with high influence can realize the rapid dissemination of information by mobilizing their huge number of followers (Asadi and Agah, 2018; Li et al., 2020a, 2020b, 2020c, 2020d; Zamparas et al., 2015).

In addition, according to the influencer theory, different users on social media have great differences in influence, and information spreads through a few influential users (Gladwell, 2006; Keller and Berry, 2003). The different influence of different types of users on information dissemination also confirms this point. For example, there are differences in information dissemination between news media and celebrities, media organizations and ordinary users, celebrities and ordinary users (Leavitt et al., 2009; Razis and Anagnostopoulos, 2014; Srinivasan et al., 2013). Although the influence of user types on information dissemination has attracted the attention of scholars, most of the previous studies are from the perspective of content, dividing user types into recipients, disseminators, and other perspectives according to content or influence types, lacking type analysis based on attribute characteristics of users (Jabeur et al., 2012; Li et al., 2014). Even if there is an analysis based on attribute characteristics, it is mostly divided into two or three categories from the perspective of disseminators, and it is impossible to make a more comprehensive analysis of users with different attribute characteristics (del Fresno Garcia et al., 2016; Riquelme and González-Cantergiani, 2016). Therefore, it is not only a supplement to the previous research, but also a further revelation of the law of information dissemination to divide users into multiple categories according to their attribute characteristics, and then explore the role of attribute characteristics in information dissemination.

Based on the existing research, we believe that user influence plays an important role in the process of information dissemination with theoretical support, and put forward the following assumptions.

H2a: In public health emergencies, the number of users’ followers has different influences on the dissemination of posts under different topic types on social media.

H2b: In public health emergencies, the number of users’ followers has different influences on the spread of posts under different sentiment types on social media.

H2c: In public health emergencies, the number of users’ followers has different influences on the spread of posts under different emotion types on social media.

H2d: In public health emergencies, different types of users have different influences on the dissemination of posts under different topic types on social media.

H2e: In public health emergencies, different types of users have different influences on the dissemination of posts under different sentiment types on social media.

H2f: In public health emergencies, different types of users have different influences on the dissemination of posts under different emotion types on social media.

### Life cycles of information dissemination

The occurrence of public health emergencies is not formed in a short time, but has gone through a series of different stages and formed different life cycles. The same is true of the dissemination dynamics of event-related information on social media, and it has different changes in different stages of life cycles (Wei and Zhang, 2019). When emergencies occur, the theory of life cycle divides the crisis brought by emergencies into multiple stages from occurrence to recession based on time trends. There are three common stages, as pre-crisis, crisis event, and post-crisis (Coombs, 2014), four stages, such as prodromal stage, outbreak stage, chronic stage, and resolution stage (Fink, 1986), and five stages, such as: pre-crisis stage, initial stage, maintenance stage, resolution stage and evaluation stage, etc. (Herovic et al., 2020). Similar to the life cycle of emergencies, the dissemination of event-related information has also gone through several life cycles, and the life cycles of public opinion are often divided by the amount of information or time trends in different stages of events. For example, the spread of network public opinion is divided into the spread period, the control period, and the stable period, and then the characteristics of network public opinion evolving in emergencies are analyzed (Li et al., 2020a, 2020b, 2020c, 2020d). Or based on the outbreak and effective control of public health emergencies, the spread of online public opinion related to events is divided into four stages, and then the analysis of public opinion in life cycles is realized (Zhang et al., 2020a, 2020b). Public opinion dissemination in different life cycles reflects the development trend of public health emergencies, and there are great differences in the dissemination of information in different stages of life cycles.

The dissemination of information in different life cycles is mainly divided into two changes. On the one hand, under different life cycles, the attraction of event topics to the public is different, and there are great differences in the topics discussed and the contents disseminated by the public on social media (An et al., 2018). For example, when a public health emergency just happens, the negative impact of the virus on holidays and life may attract more public attention. Over time, the occurrence of diseases and the harm brought by viruses to the human body have focused people’s attention, which has further shifted to the prevention and treatment with the expansion of the impact of viruses on the body (Zhang et al., 2020a, 2020b). On the other hand, under different life cycles, with the different development trends of emergencies, the spread of emotions on social media is also different (An et al., 2021). For example, studies have shown that in the initial and explosive stages of public opinion, emotions play a more obvious role in the dissemination of information, and online posts containing emotions are easier to be forwarded and disseminated by network users at this time. At the same time, in different stages of life cycles, the promotion of different emotions on information dissemination has also changed, showing different differences (Wei and Zhang, 2019). Therefore, combined with the life cycle theory, we believe that the life cycles of public opinion can affect the dissemination of posts under different topics and emotions on social media. Based on this, we put forward the following assumptions.

H3a: In public health emergencies, the life cycle of public opinion has different influences on the dissemination of posts on different topics on social media.

H3b: In public health emergencies, the life cycle of public opinion has different influences on the dissemination of posts under different sentiments on social media.

H3c: In public health emergencies, the life cycle of public opinion has different influences on the spread of posts under different emotions on social media.

## Methods

**Fig. 2:**
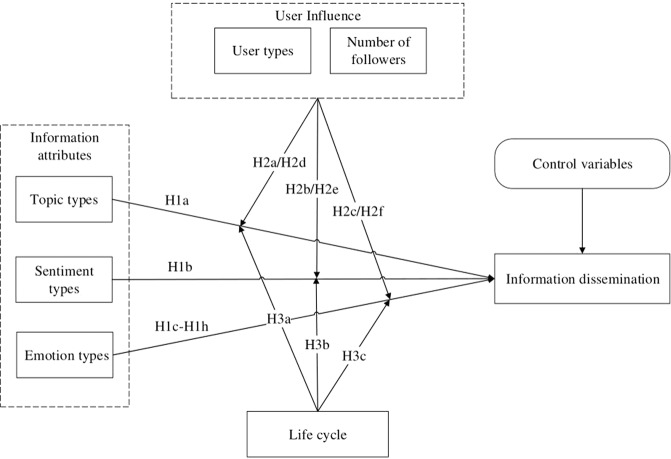
Research model. The model contains the variables analyzed and the framework of the research.

In this study, we took specifically the public health emergency as the research object to explore the factors influencing the dissemination of event-related information on social media, and then provide references and suggestions for public emergency management and network security after the occurrence of public health emergencies. In the research process, we took the topic category, sentiment category, and emotion category of posts as independent variables, and the dissemination of information as dependent variables. Moreover, the influencing factors such as user influence and life cycles were selected as moderating variables to explore the influencing mechanism behind the dissemination of posts on social media. The research model is shown in Fig. 2.

### Data collection

To test the spread of information related to public health emergencies on social media, we selected the epidemic incident of “Diamond Princess” at the beginning of the epidemic as the research object. The incident mainly occurred from the end of January to the beginning of February 2020, during which COVID-19 pneumonia spread rapidly in the closed environment of ships. According to statistics, more than 700 people on the ship were infected with COVID-19 pneumonia, accounting for about 21% of the total number of people on the ship. The reason why we chose this emergency as our research object is mainly based on the following considerations. First of all, due to the long occurrence period, the incident has received continuous reports from relevant media and continuous attention from users of social media platforms. Compared with previous studies, which observed short-term events with simple life cycles, this event can observe and collect more abundant and continuous samples, and provide more generalized conclusions. Secondly, as a large-scale public health emergency at the beginning of the COVID-19 epidemic, this incident can better reflect the public’s views and attitudes at the beginning of public health emergencies. At the same time, because the incident broke out at the beginning of the epidemic, choosing the incident can avoid the data deviation caused by reverse causality as much as possible. Finally, as an event with high influence, the network public opinion related to this emergency has attracted the attention and discussion of a large number of users, providing us with abundant data sources.

Sina Weibo was selected as our platform for obtaining data. As one of the most commonly used and influential social media platforms in China, Sina Weibo has become an important channel for the public to obtain information and discuss interactively during the epidemic, which can meet our data needs. At the same time, we collected relevant data in the form of time and keyword combinations. According to the time range of the incident, we limited the time range of data collection from January 19 to February 21, 2020. By searching for relevant keywords such as “Ship Epidemic”, “Diamond Princess epidemic” and so on, we located the discussions and topics related to the incident on the social media platform, and obtained a series of relevant data such as original posts, forwarded posts, and user information. Moreover, after completing the work of data collection, we completed the data preprocessing by browsing and deleting irrelevant posts, filtering regular expressions, and dividing the word segmentation with Jieba. Finally, we collected 79,202 posts for analysis after removing the missing values.

### Operationalization for variables

Information dissemination. As an important research object of text research on social media, information dissemination has been widely used in many studies. Existing studies often quantify information dissemination as the number of reposts and use the number of reposts to measure the popularity or diffusion trend of information (Chen et al., 2020; Li et al., 2020a, 2020b, 2020c, 2020d; Zhou et al., 2021). However, considering that the research topic is to explore the differences in information dissemination of posts under different topics and emotions when public health emergencies occur, as well as explore the specific factors affecting information dissemination. Therefore, to eliminate the interference of confounding factors, we chose the outdegree centrality of every post as the main variable to measure information dissemination. As one of the commonly used indicators of social network analysis methods, outdegree centrality can not only better measure the importance of nodes in the network, but also be widely used to analyze the dissemination of information on social media (Dey and Roy, 2017; Forouzandeh et al., 2018; Sadri et al., 2020). In terms of meaning, the outdegree centrality represents the sum of the number of connections with other nodes in the network starting from this point. Compared with degree centrality, outdegree centrality can more accurately measure the spreading scale of posts in the network. This variable was obtained through the communication network between different users. Whenever a user forwards the original post, the outdegree centrality of the post is increased by one. The outdegree centrality of different posts represents the different communication trends of posts in social networks and reflects the specific differences in information transmission. Therefore, the research can better measure the dissemination of information on social media with the help of the measurement of outdegree centrality.

#### Topic category

When analyzing the spread of posts related to public health emergencies, we first used the topic classification method to assign each post a topic tag and further compared the spread differences of posts under different topics on social media. Although traditional topic models represented by LDA are commonly used in topic modeling, LDA and its extended models show poor performance when analyzing short texts represented by texts on social media (An et al., 2021). At the same time, in the field of natural language processing, pre-training models have shown better performance and have been widely used in research fields such as topic modeling (Vayansky and Kumar, 2020). Combined with the deep learning method, this study used the Bert and Top2vec model to classify texts. Different from the traditional model, this model can automatically obtain the number of topics based on the joint semantic embedding of documents and semantic words and shows better results in topic classification (Angelov, 2020). The model mainly classifies topics through the following steps. Step 1: We first implemented the creation of the jointly embedded document and word vectors through the sentence transformer of BERT. In this process, semantically similar documents were aggregated in the vector space, and the words that bring the documents together were the topic (Griffiths et al., 2007). Step 2: Secondly, considering the sparsity of document vectors in high-dimensional space and the need to find dense document clusters more accurately and effectively, we used the UMAP method to create low-dimensional document vectors (McInnes et al., 2018), and used the HDBSCAN density clustering method to obtain dense document regions (McInnes et al., 2017). Step 3: Finally, we calculated the centroid of the obtained document dense area to generate the topic vector corresponding to the dense area, and then obtained the topic words needed for the research from near to far according to the distance from the topic vector (Angelov, 2020). Most of all, based on the topic vector and topic vocabulary of each dense area, we selected the appropriate classification standard and assigned the topic label corresponding to each dense area to classify texts.

#### Sentiment and emotion types

When public health emergencies occur, the spread of posts is often accompanied by the spread of emotions. Therefore, by dividing texts into different emotional types, we can further study the spread differences of emotional posts on social media (Steinert, 2020; Wheaton et al., 2021). Traditional emotion analysis methods are mainly divided into the methods of semantic-based emotion dictionary and machine learning-based emotion recognition (Soong et al., 2019). Recently, the research based on the deep learning model as an emotion classifier has achieved good results in classification and gradually replaced the traditional machine learning methods (Poria et al., 2017). Among them, the LSTM model that can learn long-term dependence and solve the problem of short-term memory is widely used in recognition research as the common deep learning method (Chao et al., 2015). To further improve the efficiency and accuracy of information processing, this study used the bidirectional LSTM model as the main tool for emotion classification (Li et al., 2020a, 2020b, 2020c, 2020d). The model was applied to the classification of emotions, which was mainly divided into two steps. Step 1: The word embedding model was pre-trained on a microblog data set containing 200,000 valid samples randomly crawled from Weibo to achieve better representation effects. We used the Word2vec method and trained based on the CBOW model (Mikolov et al., 2013) to get the word embedding model, which projects each word into a 100-dimensional vector space. Step 2: We connected the pre-trained word embedding model with the BiLSTM classifier and trained the emotion classification model on the data set of Weibo posts published by NLP&CC (Cheng et al., 2020; Lai et al., 2020). Using this data set, we can divide the texts into seven common emotional types: like, happiness, surprise, sadness, fear, disgust, and anger. The accuracy of the model was 0.71, which can meet the research need for emotional classification.

In addition, previous researches have tended to view pride, love, happiness, and liking as positive, while categorizing sadness, fear, nausea, and anger as negative (Moroń and Biolik-Moroń, 2021; Yang et al., 2019). After obtaining the emotion type corresponding to each microblog, to get a coarse-grained representation of the sentiment type, we divided the emotions of like and happiness into positive sentiment, while the emotions of sadness, fear, disgust and anger were divided into negative sentiment according to the corresponding relationship between sentiments and emotions (Lee and Hong, 2016).

#### User influence

As an important factor affecting the spread of posts on social media, we included user influence as a moderating variable in the research. Considering that as a standard that directly reflects the influence of users, the number of users’ followers can reflect the audience size and influence scope of users, and the difference in user types will have a greater impact on the influence of posts in the process of information dissemination (Asadi and Agah, 2018; Razis and Anagnostopoulos, 2014; Zamparas et al., 2015). Therefore, the number of followers and user types were taken as variables to measure users influence.

#### Life cycles

Similar to the development of emergencies, the dissemination of information on social media is also accompanied by the evolution of multiple life cycles. Based on the previous research, we divided life cycles in this study into four evolution periods: initial period, outbreak period, recurrent period, and recession period based on the characteristics of emergencies and the spread trend of information changing with time (An et al., 2021; Li et al., 2020a, 2020b, 2020c, 2020d; Zhang et al., 2020a, 2020b). Among them, the initial period was at the beginning of the event, and the information dissemination speed was slow and the amount of information was less. During the outbreak period, the emergency caused extensive discussion among users, which was manifested by the fast speed of information dissemination and the rapid growth of information and reached its peak. The recurrent period was the evolution stage of the event after the outbreak stage and in the subsequent process of a series of epidemic control and spread. The sudden characteristics of this period were that the speed of information was in a non-linear state and the amount of information growth had no fixed trend. In the recession period, the event gradually subsided. The speed of information and the amount of information growth gradually declined.

#### Control variables

In addition to the above main variables, we also selected several control variables to reduce the interference of confounding factors in the research. Based on the data attributes of social media, we select a series of variables such as sentence complexity, content length, URL, and hashtags as control variables and incorporate them into our research model (Table 1).

**Table 1:** Definition of variables.

Variable categories	Measure item	Description
*Dependent variable*		
Information dissemination	Inf	Number of out-degree of each post
*Independent variables*		
Topic types	Topic	The categorical variable represents the topic type of each post
Sentiment types	Sentiment	The categorical variable represents the sentiment type of each post
Emotion types	Emotion	The categorical variable represents the emotion type of each post
*Moderate variables*		
User types	User types	The categorical variable represents the user type of each post
Number of followers	Followers	Number of followers of the publisher
Life cycles	Life cycles	The categorical variable represents the life cycles of each post
*Control variables*		
Sentence complexity	Complexity	Represents the mean sentence length of each post
Content length	Length	Represents the content length of each post
URL	URL	Categorical variable, whether each post contains a URL
Hashtags	Hash	Number of hashtags of each post

### Descriptive analysis

After obtaining the data and completing the data preprocessing, we made a descriptive analysis of each variable (Table 2). Among them, Topic 1 represents posts without text content such as mood. In the follow-up study, we used it as a reference group to compare the dissemination of information under different topics on social media. Topic 2 mainly focuses on worries about the epidemic situation, topic 3 mainly focuses on posts praying for the epidemic situation, and topic 4 mainly focuses on factual information. In the data sample, we also found that posts with no text content are easier to spread on social media. From the perspective of sentiment and emotion, the information of positive sentiment and like has more distribution. In addition, the study found that posts published during the recurrent period and posts by media users were the most widely distributed in number.

**Table 2:** Descriptive statistics.

Variable	Percentage (%)/mean (S.D.)
Inf	0.768 (22.24)
*Topic*	
Topic 1	53.66
Topic 2	18.71
Topic 3	12.46
Topic 4	15.17
*Sentiment*	
Positive sentiment	71.02
Negative sentiment	28.98
*Emotion*	
Like	67.28
Disgust	9.58
Happiness	3.74
Fear	2.53
Sadness	8.13
Anger	8.73
*Life cycles*	
Initial period	1.05
Outbreak period	15.53
Recurrent period	77.24
Recession period	6.18
Followers	285,000 (3,850,000)
*User types*	
Ordinary users	85.28
Celebrities	10.48
Government	1.82
Institutions and enterprise	0.79
Media	1.63
Complexity	7.205 (7.623)
Length	15.958 (30.88)
URL	0.001 (0.032)
Hash	0.036 (0.186)

## Results

### Distribution of topics and sentiments

**Fig. 3:**
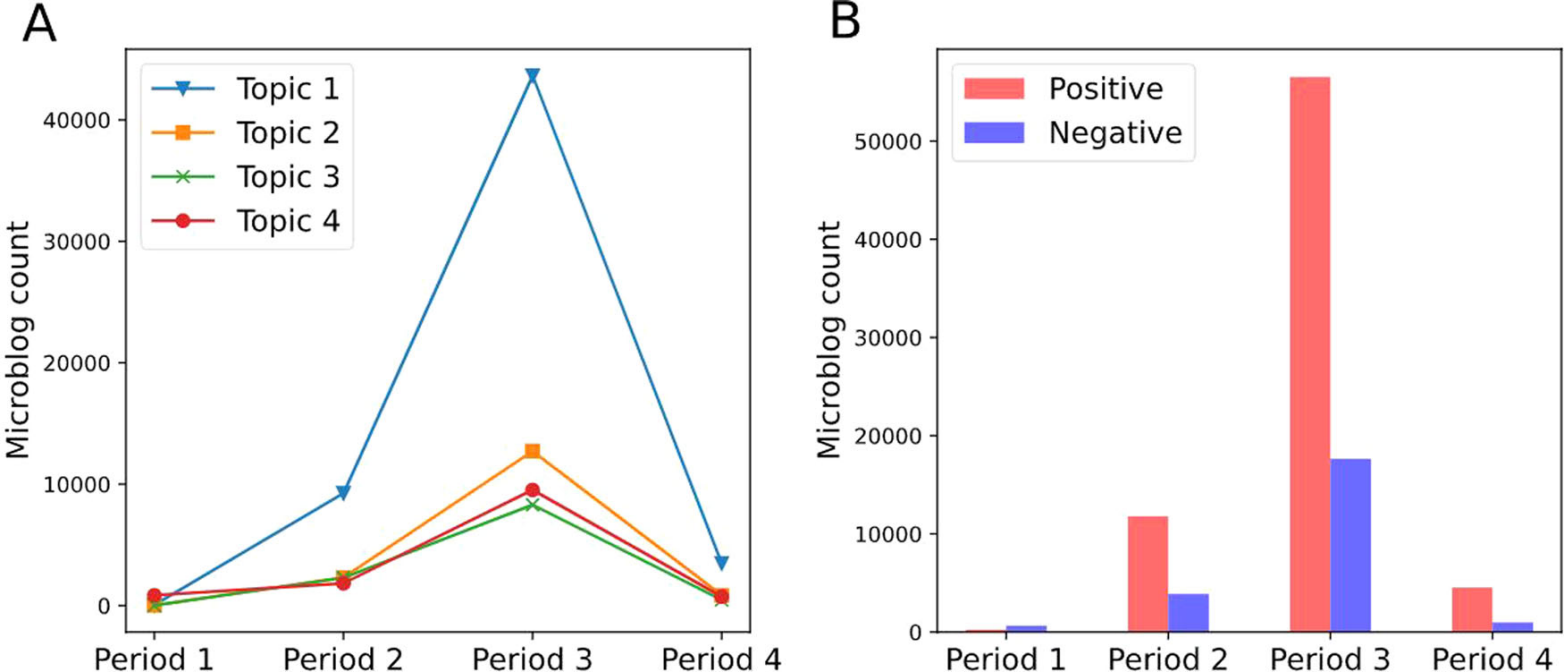
Distribution of posts with topic and sentiment types in different life cycles. **A** Distribution differences of topics in different life cycles. The horizontal axis represents the four periods of the life cycle, and the vertical axis represents the amount of information under different topics. **B** Distribution differences of sentiments in different life cycles. The horizontal axis shows the different stages of the life cycle, and the vertical axis shows the amount of information under different sentiments.

To explore the dissemination of information when public health emergencies occur from the perspective of the cognitive dimension and sentiment dimension, we draw the trend of posts with different topics and sentiments changing with the period on social media (Fig. 3). Among them, the initial period, outbreak period, recurrent period, and recession period of life cycles are represented by period 1, period 2, period 3, and period 4, respectively.

In the public health emergency, the spread of posts with different topics on social media shows an inverted “V” trend, increasing gradually in the initial period and the outbreak period, and reaching the top in the recurrent period (Fig. 3A). Subsequently, the spread speed of information gradually declined and returned to a calm state during the recession period (Fig. 3A). Among the four topic types, topic 1, within which posts have no text content, occupied a larger share on social media. Other topics of posts are relatively small and occupy a lower proportion on social media (Fig. 3A).

At the beginning of life cycles, the occurrence of public health emergencies made people have more negative sentiment, and the number of negative posts was more than that of positive posts (Fig. 3B). However, as the public opinion related to the event entered the outbreak period and the recurrent period, the number of posts was increasing, and the posts with the positive sentiment published by the public were much higher than those with negative sentiment (Fig. 3B). In the recession period, although the attention to events has decreased and the spread speed of information related to events has slowed down, the number of posts with positive sentiment was still higher than that with negative sentiment (Fig. 3B).

### Regression model

The above analysis showed that when public health emergencies occur, the distribution of relevant information on social media changes with the life cycles, and the distribution differences of the information are under different topics and sentiments. However, the above research has not yet studied the specific influence and effect of information dissemination from the perspective of topic and sentiment. Therefore, we used regression analysis to further explore the dissemination of information related to public health emergencies.

Combined with previous researches, we first carried out the logarithmic transformation on dependent variables and some covariates and used general linear regression to carry out the hierarchical tests (Chen et al., 2020). In addition, we also tested the variance inflation factor (VIF) of each variable in each model. The test results showed that the VIF values of each variable were less than the threshold of 10. Therefore, there was no serious multicollinearity problem in the study. The full measurement model is as follows:

1 $$\begin{array}{l}{\mathrm{Inf}}_i = \beta _0 + \beta _1{\mathrm{Topic}} + \beta _2{\mathrm{User}}\,{\mathrm{types}} + \beta _3{\mathrm{Followers}} \\\qquad\;\;\; +\,\beta _4{\mathrm{Lift}}\,{\mathrm{cycles}} + \beta _5{\mathrm{Topic}} \;*\; {\mathrm{User}}\,{\mathrm{types}} + \beta _6{\mathrm{Topic}} \;*\; {\mathrm{Followers}}\\\qquad\;\;\;+\,\beta _7{\mathrm{Topic}} \;*\; {\mathrm{Life}}\,{\mathrm{cycle}} + \beta _8C\end{array}$$

2 $$\begin{array}{l}{\mathrm{Inf}}_i = \beta _0 + \beta _1{\mathrm{Sentiment}} + \beta _2{\mathrm{topic}} + \beta _3{\mathrm{Usertypes}}\\\qquad\;\;\; +\,\beta _4{\mathrm{Followers}} + \beta _5{\mathrm{Liftcycles}} + \beta _6{\mathrm{Sentiment}} \;*\; {\mathrm{User}}\,{\mathrm{types}}\\\qquad\;\;\;+\, \beta _7{\mathrm{Sentiment}} \;*\; {\mathrm{Followers}} + \beta _8{\mathrm{Sentiment}} \;*\; {\mathrm{Life}}\,{\mathrm{cycle}} + \beta _9C\end{array}$$

3 $$\begin{array}{l}{\mathrm{Inf}}_i = \beta _0 + \beta _1{\mathrm{Emotion}} + \beta _2{\mathrm{Topic}} + \beta _3{\mathrm{User}}\,{\mathrm{types}}\\\qquad\;\;\;+\, \beta _4{\mathrm{Followers}} + \beta _5{\mathrm{Lift}}\,{\mathrm{cycles}} + \beta _6{\mathrm{Emotion}} \;*\; {\mathrm{User}}\,{\mathrm{types}}\\\qquad\;\;\; +\, \beta _7{\mathrm{Emotion}} \;*\; {\mathrm{Followers}} + \beta _8{\mathrm{Emotion}} \;*\; {\mathrm{Life}}\,{\mathrm{cycle}} + \beta _9C\end{array}$$

Among them, model 1 was used to explore the influence of topic types on information dissemination, model 2 was used to explore the influence of sentiment types on information dissemination, and model 3 was used to explore the influence of specific emotion types on information dissemination. Among them, user types, the number of followers, and life cycles existed as moderating variables in the three models. *C* represented control variables such as content complexity, content length, the existence of URL, and hashtag.

### Main effect model

Based on the research hypotheses, the main effect was tested by hierarchical regression (Table 3). Model 1 represented the influence of the number of followers, user types, and control variables on information dissemination. Model 2 introduced topic variables based on model 1 to explore the influence of topic types on information dissemination. Among them, the topic variable took topic 1 as the reference group. The results showed that topic 2 (*β* = −0.032, *p* < 0.001) and topic 4 (*β* = −0.102, *p* < 0.001) had a negative and significant impact on information dissemination, while topic 3 (*β* = 0.027, *p* < 0.001) had a positive and significant impact on information dissemination. Among different topic types, the information on topic 3 was easier to spread than other topics on social media. H1a was supported. To avoid the interaction between sentiment and emotion, model 3 and model 4 were established based on model 2, and the influence of sentiment and emotion on information dissemination was tested by introducing sentiment and emotion variables. Model 3 showed that the sentiment of posts (*β* = −0.009, *p* < 0.001) has a significant negative effect on information dissemination. Compared with positive sentiment, negative sentiment reduced the amount of information dissemination by 0.9% units. H1b was supported. Model 4 showed that fear (*β* = −0.022, *p* < 0.01) and sadness (*β* = −0.019, *p* < 0.001) have a negative and significant effect on information dissemination, while happiness (*β* = 0.027, *p* < 0.001) and anger (*β* = 0.012, *p* < 0.01) had a positive and significant effect on information dissemination. Among them, the regression coefficient of happiness was greater than that of anger. Compared with posts under other emotions, posts with happiness were easier to spread on social media. H1d was supported.

**Table 3:** Main effect.

Variables	DV: Ln (Inf)			
	Model 1	Model 2	Model 3	Model 4
Topic 2		−0.032*** (0.004)	−0.028*** (0.005)	−0.030*** (0.005)
Topic 3		0.027*** (0.004)	0.030*** (0.004)	0.029*** (0.004)
Topic 4		−0.102*** (0.008)	−0.097*** (0.008)	−0.098*** (0.008)
Sentiment			−0.009** (0.003)	
Disgust				−0.009 (0.005)
Happiness				0.027*** (0.006)
Fear				−0.022** (0.008)
Sadness				−0.019*** (0.005)
Anger				0.012** (0.004)
Ln (Followers)	0.037*** (0.001)	0.037*** (0.001)	0.037*** (0.001)	0.037*** (0.001)
Celebrities	−0.030*** (0.005)	−0.030*** (0.005)	−0.030*** (0.005)	−0.029*** (0.005)
Government	−0.078*** (0.009)	−0.074*** (0.009)	−0.074*** (0.009)	−0.074*** (0.009)
Institutions and enterprises	−0.013 (0.013)	−0.009 (0.013)	−0.009 (0.013)	−0.009 (0.013)
Media	0.565*** (0.012)	0.550*** (0.012)	0.550*** (0.012)	0.548*** (0.012)
Ln (Complexity)	−0.107*** (0.004)	−0.101*** (0.004)	−0.101*** (0.004)	−0.101*** (0.004)
Ln (Length)	0.092*** (0.003)	0.126*** (0.004)	0.126*** (0.004)	0.126*** (0.004)
URL	−0.421*** (0.036)	−0.424*** (0.036)	−0.424*** (0.036)	−0.428*** (0.036)
Hash	0.270*** (0.007)	0.273*** (0.008)	0.271*** (0.008)	0.270*** (0.008)
Constant	−0.162*** (0.005)	−0.227*** (0.007)	−0.228*** (0.007)	−0.228*** (0.007)
Observations	79202	79202	79202	79202
Adjusted *R*^2^	0.270	0.272	0.272	0.272

Note: Standard errors in parentheses; *p < 0.05, **p < 0.01, ***p < 0.001; Ln (*x* + 1) is denoted by Ln(*x*).

### Moderating effect of user influence

Based on the research hypotheses, we also verified the moderating effect of the number of followers (Table 4). Model 1 represents the influence of followers, user types, and control variables on information dissemination. Model 2 added topic types and interactive items of followers and topic types based on model 1. Since the topic type was a classification variable, we took topic 1 as the reference group. The results showed that the interaction items of followers and topic 2 (*β* = 0.035, *p* < 0.001), followers and topic 3 (*β* = 0.040, *p* < 0.001), and followers and topic 4 (*β* = 0.079, *p* < 0.001) were positive and significant. Therefore, the number of followers had a positive moderating effect on the relationship between topic types and information dissemination. H2a was supported. Model 3 tested the moderating effect of the number of followers on the relationship between sentiment and information dissemination. The results showed that the interaction effect between sentiment and the number of followers (*β* = 0.025, *p* < 0.001) was positive and significant. Therefore, the number of followers played a positive role in moderating the relationship between sentiments and information dissemination. H2b was supported. Model 4 examined the moderating effect of the number of followers on the influence of emotions on information dissemination. The results showed that the number of followers had positive and significant interactions with disgust (*β* = 0.038, *p* < 0.001), happiness (*β* = 0.050, *p* < 0.001), fear (*β* = 0.028, *p* < 0.001), sadness (*β* = 0.012, *p* < 0.001) and anger (*β* = 0.035, *p* < 0.001). The number of followers played a positive moderating role in the relationship between emotions and information dissemination. H2c was supported. The number of followers played a positive role in moderating the relationship between information attributes and information dissemination.

**Table 4:** Moderating effect of the number of users’ followers.

Variables	DV: Ln (Inf)			
	Model 1	Model 2	Model 3	Model 4
Topic 2		−0.183*** (0.009)	−0.010* (0.005)	−0.006 (0.005)
Topic 3		−0.191*** (0.011)	0.040*** (0.004)	0.040*** (0.004)
Topic 4		−0.524*** (0.010)	−0.079*** (0.008)	−0.072*** (0.008)
Sentiment			−0.168*** (0.007)	
Disgust				−0.259*** (0.010)
Happiness				−0.293*** (0.014)
Fear				−0.203*** (0.017)
Sadness				−0.091*** (0.012)
Anger				−0.207*** (0.010)
Ln (followers)	0.037*** (0.001)	0.009*** (0.001)	0.029*** (0.001)	0.026*** (0.001)
Topic 2 * Ln (Followers)		0.035*** (0.001)		
Topic 3 * Ln (Followers)		0.040*** (0.002)		
Topic 4 * Ln (Followers)		0.079*** (0.001)		
Sentiment * Ln (Followers)			0.025*** (0.001)	
Disgust * Ln (Followers)				0.038***
				(0.001)
Happiness * Ln (Followers)				0.050*** (0.002)
Fear * Ln (Followers)				0.028*** (0.002)
Sadness * Ln (Followers)				0.012*** (0.002)
Anger * Ln (Followers)				0.035*** (0.001)
Celebrities	−0.030*** (0.005)	−0.079*** (0.005)	−0.045*** (0.005)	−0.045*** (0.005)
Government	−0.078*** (0.009)	−0.042*** (0.009)	−0.062*** (0.009)	−0.056*** (0.009)
Institutions and enterprises	−0.013 (0.013)	−0.018 (0.013)	−0.005 (0.013)	−0.002 (0.013)
Media	0.565*** (0.012)	0.329*** (0.012)	0.521*** (0.012)	0.484*** (0.012)
Ln (Complexity)	−0.107*** (0.004)	−0.077*** (0.004)	−0.097*** (0.004)	−0.094*** (0.004)
Ln (Length)	0.092*** (0.003)	0.072*** (0.004)	0.117*** (0.004)	0.113*** (0.004)
URL	−0.421*** (0.036)	−0.576*** (0.035)	−0.458*** (0.036)	−0.502*** (0.036)
Hash	0.270*** (0.007)	0.172*** (0.008)	0.258*** (0.008)	0.241*** (0.008)
Constant	−0.162*** (0.005)	−0.027*** (0.007)	−0.178*** (0.007)	−0.158*** (0.007)
Observations	79202	79202	79202	79202
Adjusted *R*^2^	0.270	0.311	0.278	0.287

Note: Standard errors in parentheses; *p < 0.05, **p < 0.01, ***p < 0.001.

**Fig. 4:**
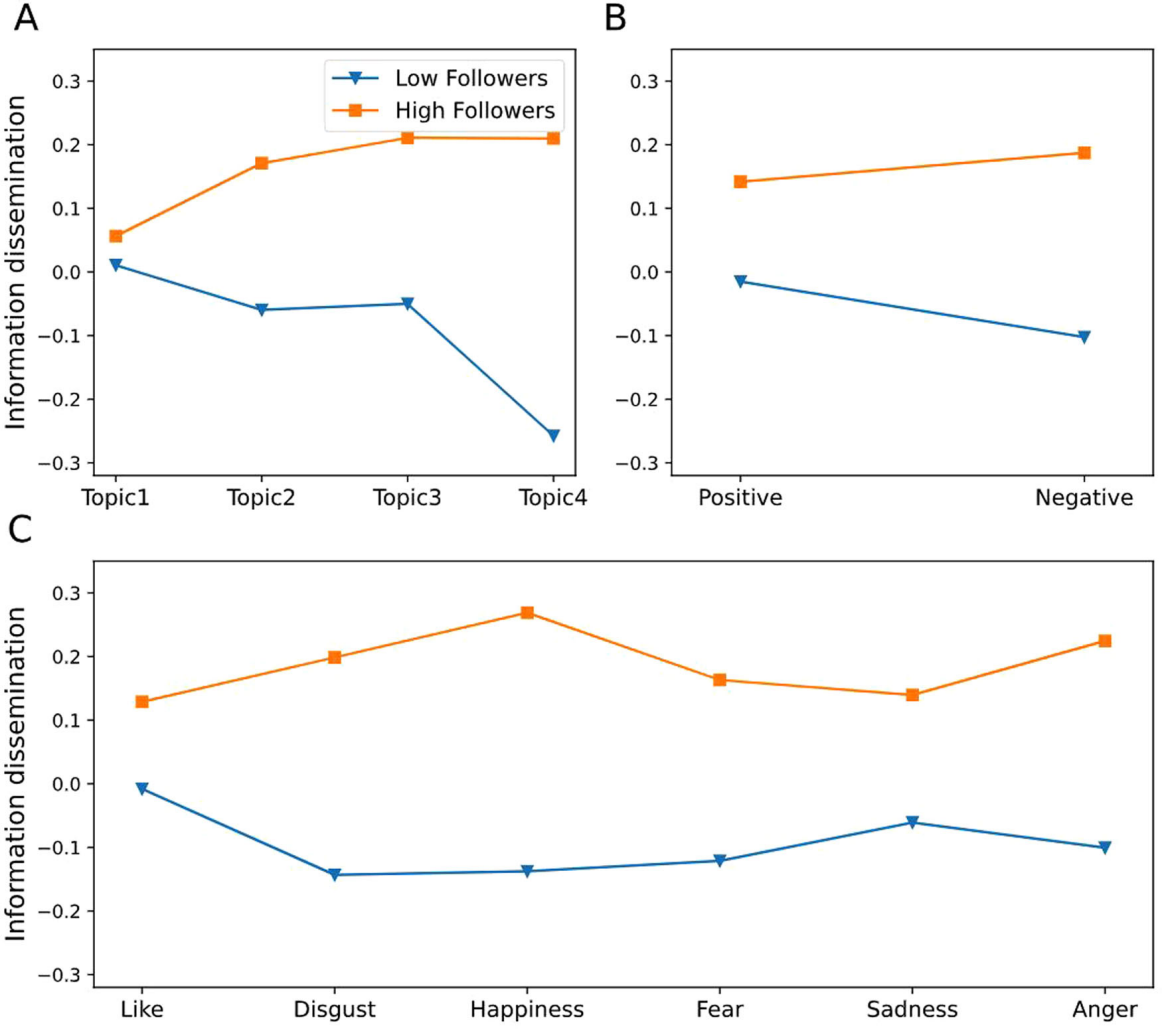
The moderating effect of the number of followers on topic, sentiment, and emotion types. **A** Moderating effect of followers on topics. The horizontal axis represents different topic types, and the vertical axis represents the spread of information on social media. **B** Moderating effects of followers on sentiments. The horizontal axis shows information that contains positive or negative sentiment. **C** Moderating effects of followers on emotions. The horizontal axis shows posts containing different emotions, such as like, anger, and sadness.

To further understand the moderating role of the number of followers in the relationship between information attributes and information dissemination, we have drawn the graphs of moderating effect based on previous researches (Fig. 4) (Chen et al., 2020). Except for topic types without text content, the difference between the spread of posts published by users with different numbers of followers was not obvious. However, among other types of topics, there were significant differences in the dissemination of posts published by users with different numbers of followers. Especially in factual information, the difference between users with different numbers of followers on information dissemination was particularly obvious (Fig. 4A). At the same time, it can also be found that under different sentiment types (Fig. 4B) and emotion types (Fig. 4C), there are also significant differences in the dissemination of posts published by users with different numbers of followers on social media. This difference existed significantly in the information dissemination under the negative sentiment, the emotions of happiness and anger. The number of followers played a positive role in the relationship between information attributes and information dissemination.

In addition, considering that independent variables and moderator variables are classified variables. Based on controlling a series of variables, this paper explored the moderating effect of user types through a multivariate analysis of variance (Table 5). Among them, model 1 tested the moderating role of user type in the relationship between topic type and information dissemination. The results showed that the main effects of topic types (*F* = 105.39, *p* < 0.001), user types (*F* = 23.54, *p* < 0.001) and their interaction items (*F* = 128.68, *p* < 0.001) were significant. Therefore, user types played a moderating role in the relationship between topic types and information dissemination. H2d was supported. Model 2 tested the role of user types in the relationship between sentiment types and information dissemination based on controlling topic types. The results showed that the main effect of sentiment types (*F* = 174.49, *p* < 0.001), user types (*F* = 739.7, *p* < 0.001) and the interaction items of sentiment types and user types (*F* = 72.67, *p* < 0.001) were all significant. Therefore, user types played a moderating role in the relationship between sentiment types and information dissemination. H2e was supported. Model 3 tested the role of user types in the relationship between emotion types and information dissemination based on controlling topic types. The results showed that the main effect of emotion types (*F* = 70.81, *p* < 0.001) user types (*F* = 664.28, *p* < 0.001) and the interaction items between emotion types and user types (*F* = 40.88, *p* < 0.001) were all significant. Therefore, user types played a moderating role in the relationship between emotion types and information dissemination. H2f was supported. In a word, user types have been proven to play a moderating role in the relationship between information attributes and information dissemination.

**Table 5:** Moderating effect of users’ type.

Source	Partial SS	df	MS	*F*	Prob > *F*
**Model 1**	3231.883	24	134.662	1320.02	0.000
Topic	32.254	3	10.751	105.39	0.000
User types	9.606	4	2.402	23.54	0.000
Topic * User types	157.534	12	13.128	128.68	0.000
Ln (Followers)	348.218	1	348.218	3413.40	0.000
Ln (Complexity)	47.117	1	47.117	461.87	0.000
Ln (Length)	83.510	1	83.510	818.60	0.000
URL	17.737	1	17.737	173.86	0.000
Hash	68.079	1	68.079	667.35	0.000
Residual	8077.252	79177	0.102		
Total	11309.135	79,201	0.143		
**Model 2**	3105.325	17	182.666	1763.11	0.000
Sentiment	18.078	1	18.078	174.49	0.000
User types	306.545	4	76.636	739.7	0.000
Sentiment * User types	30.116	4	7.529	72.67	0.000
Topic	21.690	3	7.230	69.79	0.000
Ln (Followers)	369.872	1	369.872	3570.04	0.000
Ln (Complexity)	56.417	1	56.417	544.54	0.000
Ln (Length)	114.735	1	114.735	1107.44	0.000
URL	15.930	1	15.930	153.76	0.000
Hash	111.203	1	111.203	1073.34	0.000
Residual	8203.810	79,184	0.104		
Total	11309.135	79,201	0.143		
**Model 3**	3165.292	37	85.548	831.59	0.000
Emotion	36.424	5	7.285	70.81	0.000
User types	273.345	4	68.336	664.28	0.000
Emotion * User types	84.113	20	4.206	40.88	0.000
Topic	20.362	3	6.787	65.98	0.000
Ln (Followers)	361.321	1	361.321	3512.3	0.000
Ln (Complexity)	53.052	1	53.052	515.7	0.000
Ln (Length)	112.222	1	112.222	1090.88	0.000
URL	17.872	1	17.872	173.73	0.000
Hash	97.650	1	97.650	949.23	0.000
Residual	8143.843	79,164	0.103		
Total	11309.135	79,201	0.143		

*SS* denotes sum of squares of deviation from mean, *df* denotes the degree of freedom; *MS* denotes mean square, which is equal to the corresponding SS divided by the df, *F* denotes the *F* statistic, which is the statistic used for hypothesis testing in analysis of variance.

**Fig. 5:**
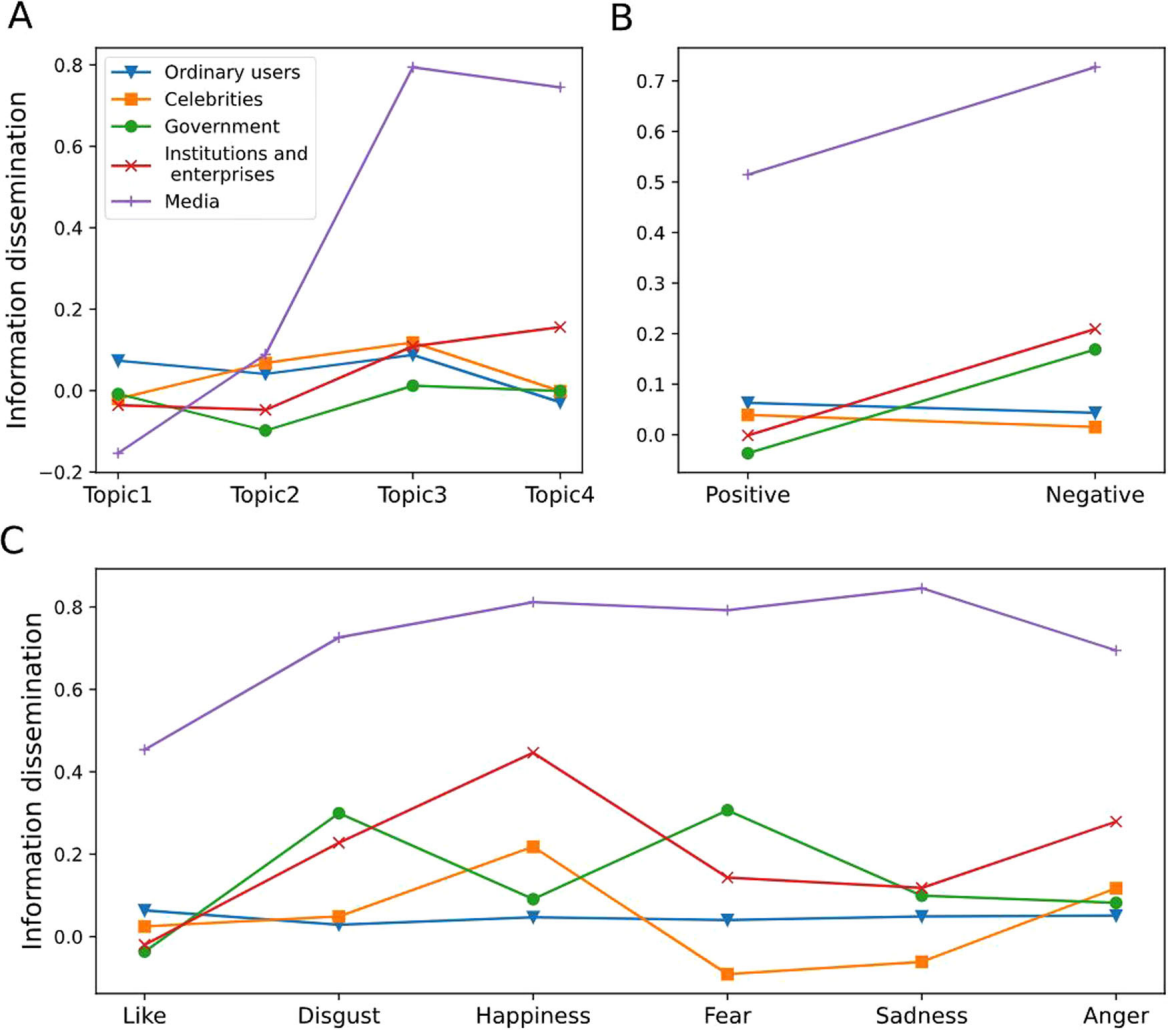
The moderating effect of user types on topic, sentiment, and emotion types. **A** Moderating effect of user types on topics. The horizontal axis represents information containing content of different topics, and the vertical axis represents the dissemination of information on social media. **B** Moderating effect of user types on sentiments. The horizontal axis shows information under the positive or negative sentiment. **C** Moderating effect of user types on emotions. The horizontal axis shows posts with different emotions.

In addition, we also explored the moderating role of user types by drawing graphs (Fig. 5). Compared with other types of users, information published by media users containing prayer topics and fact topics was easier to spread on social media, and this difference was significant (Fig. 5A). At the same time, for information containing different sentiment types (Fig. 5B) and emotion types (Fig. 5C), it was easier for information from media users to spread than other types of users on social media. In a word, there were significant differences in the dissemination of posts with information attributes published by different users on social media, and this difference was more obvious between media users and other types of users.

### Moderating effect of life cycles

Based on controlling a series of variables, the study also tested the role of life cycles in the relationship between information attributes and information dissemination by multivariate analysis of variance (Table 6). Among them, model 1 tested the role of life cycles in the relationship between topic types and information dissemination. The results showed that the main effect of topic types (*F* = 36.67, *p* < 0.001), the life cycle (*F* = 9.91, *p* < 0.001) and the interaction between the two items (*F* = 8.95, *p* < 0.001) were all significantly related. Therefore, life cycles played a moderating role in the relationship between topic types and information dissemination. H3a was supported. Model 2 examined the role of life cycles in the relationship between sentiment types and information dissemination. The results showed that the main effect of sentiment types (*F* = 19.27, *p* < 0.001),life cycles (*F* = 107.4, *p* < 0.001) and the interaction between them (*F* = 14.58, *p* < 0.001) were all significant. The results confirmed the moderating role of life cycles in the relationship between sentiment types and information dissemination. H3b was supported. Model 3 examined the role of life cycles in the relationship between emotion types and information dissemination. The results showed that the main effect of emotion types (*F* = 7.95, *p* < 0.001), life cycles (*F* = 69.13, *p* < 0.001) and the interaction between the two items (*F* = 4.73, *p* < 0.001) were all significant. Therefore, life cycles also played a moderating role in the relationship between emotion types and information dissemination. H3c was supported. All in all, life cycles played a moderating role in the relationship between information attributes and information dissemination.

**Table 6:** Moderating effect of life cycles.

Source	Partial SS	df	MS	*F*	Prob > *F*
**Model 1**	3129.582	24	130.399	1262.25	0.000
Topic	11.366	3	3.789	36.67	0.000
Life cycles	3.071	3	1.024	9.91	0.000
Topic * Life cycles	8.317	9	0.924	8.95	0.000
User types	292.157	4	73.039	707.01	0.000
Ln (Followers)	381.527	1	381.527	3693.13	0.000
Ln (Complexity)	52.938	1	52.938	512.43	0.000
Ln (Length)	125.844	1	125.844	1218.16	0.000
URL	12.221	1	12.221	118.29	0.000
Hash	114.494	1	114.494	1108.29	0.000
Residual	8179.553	79,177	0.103		
Total	11309.135	79,201	0.143		
**Model 2**	3126.481	19	164.552	1592.34	0.000
Sentiment	1.991	1	1.991	19.27	0.000
Life cycles	33.297	3	11.099	107.4	0.000
Sentiment * Life cycles	4.520	3	1.507	14.58	0.000
Topic	21.025	3	7.008	67.82	0.000
User types	293.052	4	73.263	708.95	0.000
Ln (Followers)	382.155	1	382.155	3698.04	0.000
Ln (Complexity)	52.739	1	52.739	510.35	0.000
Ln (Length)	125.598	1	125.598	1215.39	0.000
URL	12.304	1	12.304	119.06	0.000
Hash	112.481	1	112.481	1088.46	0.000
Residual	8182.654	79,182	0.103		
Total	11309.135	79,201	0.143		
**Model 3**	3134.732	35	89.564	867.39	0.000
Emotion	4.102	5	0.820	7.95	0.000
Life cycles	21.414	3	7.138	69.13	0.000
Emotion * Life cycles	7.325	15	0.488	4.73	0.000
Topic	20.125	3	6.708	64.97	0.000
User types	290.710	4	72.678	703.85	0.000
Ln (Followers)	380.893	1	380.893	3688.8	0.000
Ln (Complexity)	52.314	1	52.314	506.64	0.000
Ln (Length)	125.321	1	125.321	1213.69	0.000
URL	12.579	1	12.579	121.82	0.000
Hash	111.083	1	111.083	1075.8	0.000
Residual	8174.403	79,166	0.103		
Total	11309.135	79,201	0.143		

**Fig. 6:**
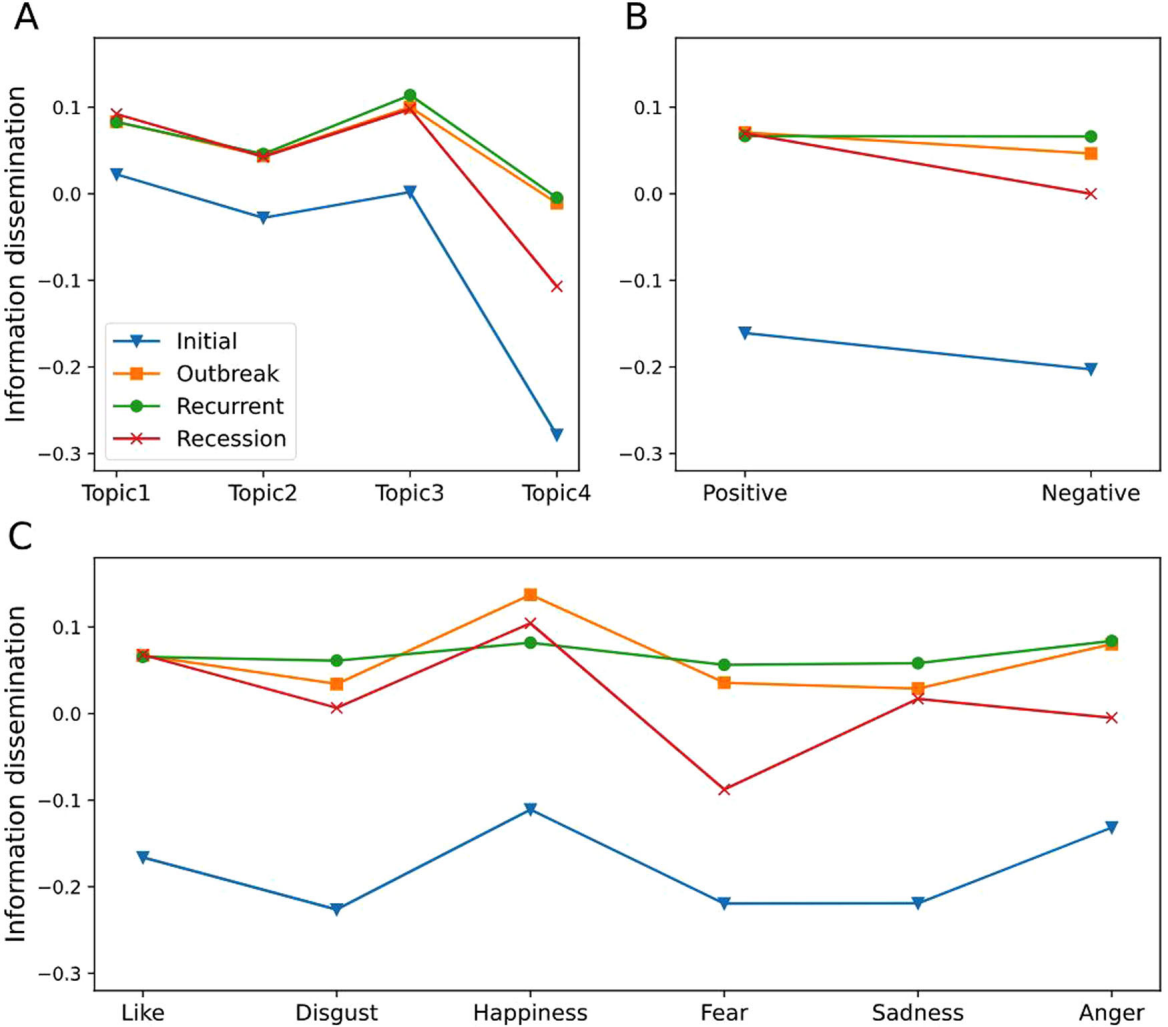
The moderating effect of life cycles on topic, sentiment, and emotion types. **A** Moderating effect of life cycles on topic. The horizontal axis represents the different topic types, and the vertical axis represents the dissemination of information. **B** Moderating effect of life cycles on topic. The horizontal axis shows information under the positive or negative sentiment. **C** Moderating effect of life cycles on topic. The horizontal axis shows different emotion types.

Figure 6 shows the moderating role of life cycles in the relationship between information attributes and information dissemination. As can be seen from Fig. 6, compared with other periods of life cycles, posts containing topic types published in the initial stage were more difficult to spread on social media. This rule was also applicable to the spread of posts under different sentiment types and emotion types. At the same time, compared with other periods of life cycles, posts with topic types published in the outbreak and recurrent periods were generally easier to spread on social media, especially for topic types with prayers and facts. In addition, after the emergency passed the initial stage, the difference in information transmission was more reflected in negative sentiment or emotion types such as fear and anger. It makes us further explored the differentiated moderating role of different periods of life cycles in the relationship between information attributes and information transmission.

### Robustness test

To test the robustness of the results, we re-encode the variables of sentiment and emotion. For example, the emotion of surprise was added to the emotion variables and neutral sentiment was added to the sentiment variables, and tested the research hypotheses based on the expanded sample size. The test results were only different in regression coefficient, and other results were similar to the test results, which proved the robustness of our research conclusions (Tables 7–10).

**Table 7:** Main effect.

Variables	DV: Ln (Inf)			
	Model 1	Model 2	Model 3	Model 4
Topic 2		−0.032*** (0.004)	−0.028*** (0.005)	−0.029*** (0.005)
Topic 3		0.025*** (0.004)	0.029*** (0.004)	0.027*** (0.004)
Topic 4		−0.104*** (0.008)	−0.099*** (0.008)	−0.100*** (0.008)
Negative sentiment			−0.010** (0.003)	
Neutral sentiment			0.003 (0.005)	
Disgust				−0.011* (0.005)
Surprise				0.005 (0.005)
Happiness				0.027*** (0.007)
Fear				−0.023** (0.008)
Sadness				−0.020*** (0.005)
Anger				0.011* (0.005)
Ln (Followers)	0.038*** (0.001)	0.038*** (0.001)	0.038*** (0.001)	0.038*** (0.001)
Celebrities	−0.031*** (0.005)	−0.031*** (0.005)	−0.031*** (0.005)	−0.030*** (0.005)
Government	−0.082*** (0.009)	−0.079*** (0.009)	−0.079*** (0.009)	−0.079*** (0.009)
Institutions and enterprises	−0.013 (0.013)	−0.009 (0.013)	−0.010 (0.013)	−0.010 (0.013)
Media	0.547*** (0.011)	0.533*** (0.011)	0.532*** (0.011)	0.531*** (0.011)
Ln (Complexity)	−0.110*** (0.004)	−0.103*** (0.004)	−0.104*** (0.004)	−0.103*** (0.004)
Ln (Length)	0.093*** (0.003)	0.128*** (0.004)	0.128*** (0.004)	0.128*** (0.004)
URL	−0.426*** (0.035)	−0.428*** (0.035)	−0.429*** (0.035)	−0.431*** (0.035)
Hash	0.275*** (0.007)	0.277*** (0.007)	0.275*** (0.008)	0.274*** (0.008)
Constant	−0.165*** (0.005)	−0.231*** (0.007)	−0.232*** (0.007)	−0.233*** (0.007)
Observations	83784	83784	83784	83784
Adjusted *R*^2^	0.277	0.279	0.279	0.279

Note: Standard errors in parentheses; ^*^p < 0.05, ^**^p < 0.01, ^***^p < 0.001.

**Table 8:** Moderating effect of the number of users’ followers.

Variables	DV: ln(Inf)			
	Model 1	Model 2	Model 3	Model 4
Topic 2		−0.186*** (0.009)	−0.011* (0.005)	−0.009 (0.005)
Topic 3		−0.199*** (0.011)	0.037*** (0.004)	0.037*** (0.004)
Topic 4		−0.531*** (0.010)	−0.083*** (0.008)	−0.077*** (0.008)
Negative sentiment			−0.167*** (0.007)	
Neutral sentiment			−0.111*** (0.012)	
Disgust				−0.258*** (0.010)
Surprise				−0.142*** (0.012)
Happiness				−0.292*** (0.015)
Fear				−0.201*** (0.018)
Sadness				−0.089*** (0.012)
Anger				−0.206*** (0.010)
Ln (Followers)	0.038*** (0.001)	0.009*** (0.001)	0.030*** (0.001)	0.026*** (0.001)
Topic 2 * Ln (Followers)		0.035*** (0.001)		
Topic 3 * Ln (Followers)		0.041*** (0.002)		
Topic 4 * Ln (Followers)		0.079*** (0.001)		
Negative * Ln (Followers)			0.025*** (0.001)	
Neutral * Ln (Followers)			0.019*** (0.002)	
Disgust * Ln (Followers)				0.038*** (0.001)
Surprise * Ln (Followers)				0.024*** (0.002)
Happiness * Ln (Followers)				0.050*** (0.002)
Fear * Ln (Followers)				0.028*** (0.002)
Sadness * Ln (Followers)				0.012*** (0.002)
Anger * Ln (Followers)				0.035*** (0.001)
Celebrities	−0.031*** (0.005)	−0.080*** (0.005)	−0.045*** (0.005)	−0.046*** (0.005)
Government	−0.082*** (0.009)	−0.045*** (0.009)	−0.067*** (0.009)	−0.061*** (0.009)
Institutions and enterprises	−0.013 (0.013)	−0.021 (0.013)	−0.007 (0.013)	−0.004 (0.013)
Media	0.547*** (0.011)	0.311*** (0.012)	0.501*** (0.011)	0.469*** (0.011)
Ln (Complexity)	−0.110*** (0.004)	−0.080*** (0.004)	−0.100*** (0.004)	−0.097*** (0.004)
Ln (Length)	0.093*** (0.003)	0.075*** (0.004)	0.119*** (0.004)	0.116*** (0.004)
URL	−0.426*** (0.035)	−0.578*** (0.034)	−0.460*** (0.035)	−0.500*** (0.034)
Hash	0.275*** (0.007)	0.175*** (0.007)	0.262*** (0.007)	0.246*** (0.007)
Constant	−0.165*** (0.005)	−0.026*** (0.007)	−0.178*** (0.007)	−0.158*** (0.007)
Observations	83784	83784	83784	83784
Adjusted *R*^2^	0.277	0.317	0.285	0.292

**Table 9:** Moderating effect of users’ type.

Source	Partial SS	df	MS	*F*	Prob > *F*
**Model 1**	3691.204	24	153.800	1437.180	0.000
Topic	30.015	3	10.005	93.490	0.000
User types	8.158	4	2.039	19.060	0.000
Topic * User types	162.223	12	13.519	126.320	0.000
Ln (Followers)	392.509	1	392.509	3667.780	0.000
Ln (Complexity)	54.445	1	54.445	508.760	0.000
Ln (Length)	95.128	1	95.128	888.920	0.000
URL	19.920	1	19.920	186.140	0.000
Hash	79.461	1	79.461	742.520	0.000
Residual	8963.492	83,759	0.107		
Total	12654.696	83,783	0.151		
**Model 2**	3561.3577	22	161.880	1491.12	0.000
Sentiment	17.625	2	8.812	81.170	0.000
User types	235.251	4	58.813	541.740	0.000
Sentiment * User types	31.155	8	3.894	35.870	0.000
Topic	23.338	3	7.779	71.660	0.000
Ln (Followers)	413.840	1	413.840	3811.990	0.000
Ln (Complexity)	64.400	1	64.400	593.200	0.000
Ln (Length)	129.196	1	129.196	1190.060	0.000
URL	18.320	1	18.320	168.750	0.000
Hash	125.498	1	125.498	1156.000	0.000
Residual	9093.339	83,761	0.109		
Total	12654.696	83,783	0.151		
**Model 3**	3,620.691	42	86.207	799.100	0.000
Emotion	35.676	6	5.946	55.120	0.000
User types	291.409	4	72.852	675.310	0.000
Emotion * User types	84.524	24	3.522	32.650	0.000
Topic	22.020	3	7.340	68.040	0.000
Ln (Followers)	405.173	1	405.173	3,755.770	0.000
Ln (Complexity)	61.085	1	61.085	566.230	0.000
Ln (Length)	126.891	1	126.891	1,176.220	0.000
URL	20.249	1	20.249	187.700	0.000
Hash	111.718	1	111.718	1,035.570	0.000
Residual	9,034.005	83,741	0.108		
Total	12,654.696	83,783	0.151

**Table 10:** Moderating effect of life cycles.

Source	Partial SS	df	MS	*F*	Prob > *F*
**Model 1**	3589.060	24	149.544	1381.66	0.000
Topic	13.017	3	4.339	40.09	0.000
Life cycles	3.461	3	1.154	10.66	0.000
Topic * Life cycles	8.848	9	0.983	9.08	0.000
User types	311.685	4	77.921	719.93	0.000
Ln (Followers)	427.917	1	427.917	3953.6	0.000
Ln (Complexity)	59.607	1	59.607	550.72	0.000
Ln (Length)	138.794	1	138.794	1282.34	0.000
URL	14.425	1	14.425	133.27	0.000
Hash	126.625	1	126.625	1169.91	0.000
Residual	9065.636	83,759	0.108		
Total	12654.696	83,783	0.151		
**Model 2**	3586.423	23	155.931	1440.28	0.000
Sentiment	2.388	2	1.194	11.03	0.000
Life cycles	24.310	3	8.103	74.85	0.000
Sentiment * Life cycles	5.132	6	0.855	7.9	0.000
Topic	21.914	3	7.305	67.47	0.000
User types	312.269	4	78.067	721.08	0.000
Ln (Followers)	427.583	1	427.583	3949.42	0.000
Ln (Complexity)	59.456	1	59.456	549.18	0.000
Ln (Length)	138.699	1	138.699	1281.1	0.000
URL	14.656	1	14.656	135.37	0.000
Hash	124.572	1	124.572	1150.63	0.000
Residual	9068.273	83,760	0.108		
Total	12654.696	83,783	0.151		
**Model 3**	3594.704	39	92.172	851.97	0.000
Emotion	4.524	6	0.754	6.97	0.000
Life cycles	25.215	3	8.405	77.69	0.000
Emotion * Life cycles	7.936	18	0.441	4.08	0.000
Topic	20.995	3	6.998	64.69	0.000
User types	310.041	4	77.510	716.45	0.000
Ln (Followers)	426.339	1	426.339	3940.77	0.000
Ln (Complexity)	59.040	1	59.040	545.72	0.000
Ln (Length)	138.508	1	138.508	1280.27	0.000
URL	14.941	1	14.941	138.1	0.000
Hash	123.042	1	123.042	1137.31	0.000
Residual	9059.992	83,744	0.108		
Total	12654.696	83,783	0.151		

## Discussion

### Main findings

The research took public emergencies as the research object and focused on information dissemination from the cognitive dimension and sentiment dimension. The main findings of the study are as follows. First, based on the theory of planned behavior, it was found that in public health emergencies, the content of information will affect the behavior of users. Among them, different topics had different influences on information dissemination, and information containing prayers was more easily forwarded and disseminated by users than other topic types. During the spread of the epidemic, the public’s acceptance of epidemic information was closely related to their risk perception. Under the influence of risk perception, the public hoped to get through the epidemic as soon as possible and reduced their risk of being infected by the epidemic (Adiyoso and Wilopo, 2021). Therefore, the information about praying to overcome the epidemic and overcome the difficulties brought by the epidemic can spread rapidly on social media. At the same time, due to openness and high interactivity, users on social media often spontaneously forward relevant information under group emotions. However, when the information content of prayers is rapidly spread, the characteristics of social media will further promote the spread of this topic type (Kwon et al., 2016).

Secondly, based on the theory of sentiment contagion, it was found that posts containing positive sentiment can spread more quickly in public health emergencies. At the same time, the study further divided sentiments into different emotions and compared the differences in information dissemination from the perspective of emotional types. The results once again confirmed that positive sentiment has a more effective role in promoting information dissemination, and posts with happiness can be spread faster than other emotions on social media. This conclusion is a verification of the Pollyanna hypothesis and is also consistent with the conclusions of previous studies (Lu et al., 2021). Compared with information containing negative emotions, the public prefers to forward information with happy and optimistic emotions that can make people feel happy (Jin et al., 2017; Liu et al., 2020). This behavior can also reduce the information pressure and relieve negative emotions such as anxiety and worry (Zhou et al., 2021).

Thirdly, it was found that the number of followers and user types played a moderating role in the relationship between information attributes and information dissemination. Based on the resource mobilization theory, it was found that posts with different topics, sentiments, and emotions published by users with high followers are more likely to be forwarded and disseminated by other users. Among them, the promotion of a high number of followers was more obvious in the dissemination of posts under factual content, negative sentiment, and happy emotion. This shows that, as one of the main measures of user influence, users with high followers have a stronger ability to mobilize social resources, and posts published on social media are more likely to attract public attention and spread (Hu et al., 2019). At the same time, based on the influencer theory, the study also found that different user types have different influences on the dissemination of information. Among them, the role that media can play was significantly different from other users. This difference was particularly obvious in the context of prayer, fact, and the information dissemination of posts under different sentiments and emotions. As one of the main channels of information dissemination, media has a higher influence on social media. In the process of information dissemination, users with higher influence have a greater effect on the dissemination of information under different topic types, sentiments, and emotions (Riquelme and González-Cantergiani, 2016).

Finally, the study also found that life cycles played a moderating role in the relationship between information attributes and information dissemination. The research confirmed the important role of life cycles and is a verification and supplement to the previous research (An et al., 2021). At the same time, based on the life cycles theory, the study also found that there were differences in the influence of information attributes on information dissemination in the four periods of life cycles. In the initial period, information attributes such as topic type, sentiment, and emotion have a weak influence on communication behavior. During the outbreak period and recurrent period, information attributes had a strong influence on information dissemination. This role was more obviously reflected in the content of prayers and facts, and the information dissemination under the negative sentiment, the emotions of fear and anger. It may be because the public paid less attention to the event-related information during the initial period, which has not enabled the information of emergency to be paid more attention to and discussed. In the outbreak period and recurrent period, public health emergencies have caused a large-scale impact, attracting a lot of attention and discussion, and posts containing different topic types, sentiments, and emotions have also been produced in large numbers and widely spread. Therefore, the role of different topic types, sentiments, and emotions has been strengthened during the outbreak period and recurrent period (Zhang et al., 2020a, 2020b).

### Theoretical implications

This study took public health emergencies as the research object and made a further study on the dissemination behavior of information related to public health emergencies on social media. First, although information dissemination has become an important research object, most of the existing studies start from a single dimension and explore the influencing factors from the cognitive dimension or sentiment dimension (Ferrara and Yang, 2015b; Wang et al., 2016; Wei and Zhang, 2019). This study took public health emergencies as the research object and made a further study on the dissemination behavior related to public health emergencies on social media. Therefore, we started from the cognitive dimension and sentiment dimension, and used deep learning methods to generate the variables of topics, sentiments, and emotions. Moreover, we used hierarchical regression to explore the influence of information attributes on information dissemination, which is a further verification and supplement to the existing research.

Secondly, when emergencies occur, most of the existing studies start from the direct influencing factors of information dissemination and explore the impact from various angles such as information content and emotion (Garcia and Rimé, 2019; Steinert, 2020). However, few studies have explored the conditional mechanism of information dissemination on social media. Therefore, based on large-scale social media data, this study incorporated media factors and time factors into the analysis, and further analyzed the conditional effects of user types, followers, and life cycles on information transmission, further revealing the transmission path and influencing factors of information. It enriches the relevant research on the influencing conditions of information dissemination on social media.

Thirdly, this study analyzed the influence of information attributes on information dissemination from the perspective of user influence. On the one hand, the analysis of user influence in existing studies only starts from the quantitative dimensions such as the number of followers or the number of posts and analyzes the impact brought by user influence. There are also studies that use user influence as a moderating variable to analyze the relationship between information attributes of a single dimension and information transmission (Li et al., 2020a, 2020b, 2020c, 2020d; Zamparas et al., 2015). However, in addition to numerical variables such as the number of followers, the impact of different user types on information dissemination is also quite different (del Fresno Garcia et al., 2016; Razis and Anagnostopoulos, 2014). Therefore, based on existing research, this study used two dimensions: followers and user types to explore the effect of users influence on information dissemination. At the same time, this study also expanded information attributes into topic types, sentiment types, and emotion types, and further explored the role of user influence between multi-dimensional information attributes and information dissemination. The findings are helpful to further explore the influence of user influence on information dissemination, and further understand how user influence plays a role in multi-dimensional information attributes and information dissemination.

Finally, this study analyzed the influence of information attributes on information dissemination from the perspective of life cycles. The occurrence of emergencies is often accompanied by different life cycles, and the dissemination of event-related information is also accompanied by multiple stages of life cycles (Wei and Zhang, 2019). Studies have confirmed that when emergencies occur, life cycles are an important factor affecting the dissemination of information on social media (Zhang et al., 2020a, 2020b). However, most of the existing studies start from the direct impact of life cycles, and few studies explore the conditional role of life cycles in the process of information dissemination (An et al., 2021; Zhang et al., 2020a, 2020b). Therefore, this study took life cycles as a moderator variable to further explore the differences in the influence of topic types, sentiments, and emotions on information dissemination in different periods. Research findings not only improve the relevant research on the life cycles in emergencies, but also make contributions to further explore the relevant conditions of information transmission on social media.

### Practical implications

The related findings also have certain practical significance. First, it is found that different topic types, sentiments, and emotion types have different influences on information dissemination. This provides a reference for us to predict, manage and control the network public opinion after public health emergencies. At the same time, through targeted guidance of some contents and emotions of public posts, public opinion can be kept in a stable or positive state more efficiently, and the negative impact brought by network mass incidents can be reduced.

Secondly, the study also found the influence of user influence on information dissemination. Posts published by users with high followers and media users can be spread more quickly. This provides suggestions for us to realize public opinion governance related to emergencies. When a public health emergency occurs, the management department can not only spread positive information through users with high followers or media users but also reduce the psychological pressure and relieve negative emotions of the public. In addition, users with a high number of followers and media users can be encouraged to make positive statements related to the event and dispel rumors to achieve more rapid and accurate control, thus reducing the negative impact of online public opinion.

Finally, from the perspective of life cycles, the study also found that the outbreak period and recurrent period were the fastest periods for information dissemination with different attributes, which provides a better intervention point of time for emergency management to control public opinion. When public health emergencies occur, the management department can intervene at the time point before the public opinion of the incident is uncontrollable, to effectively maintain the stability of the network order and realize good social governance.

### Impact on the global recovery

During the COVID-19 epidemic, the virus has physically spread in different time and space, not only causing disruption to the normal social order but also seriously affecting the health and daily life of the public. However, in addition to the impact of virus infection, the information epidemic is affecting all aspects of society and the public from many aspects. For example, real-life connections create conditions for the spread of information on the Internet. Information epidemic can spread rapidly through friends, partners, allies and even competitors, and then spread to the entire network, bringing great challenges to the network security (Li et al., 2021). Both the damage caused by disinformation to epidemic control and the impact of misinformation on public mental health force us to further study the dissemination process of information in social networks during the COVID-19 pandemic. On the one hand, understanding information dissemination under the COVID-19 epidemic can help emergency management departments to guide online public opinions more quickly in the face of similar events and reduce the negative impact brought by the information epidemic. On the other hand, although great progress has been made in COVID-19 control, we still cannot predict the exact time when the public health emergency will be completely resolved. Therefore, understanding the behavior of information dissemination under the epidemic situation can help us further realize the prevention of online mass incidents, and is more conducive to the governance of information and public opinions.

Global recovery depends not only on the development of medicines to combat the epidemic from a medical perspective but also on controlling the information epidemic and reducing its negative impact. Therefore, this paper investigates the influencing factors of information transmission to create favorable conditions for the further advancement of global recovery. Firstly, the deep learning methods were used to extract the topic, sentiment, and emotion of information content under the epidemic, and then to understand the attribute characteristics of the information under the influence of COVID-19. Secondly, the study also explores the differences from the perspectives of topic, sentiment, and emotion, to explore what kind of information is more easily spread in social networks and understand the characteristics of information dissemination under the influence of the epidemic. Finally, the study added the user factor and time factor as the moderating variables affecting information dissemination, to explore the difference of information dissemination under user influence and life cycles, and understand the influencing factors of information dissemination differentiation under the influence of the epidemic. Therefore, the research findings are conducive to the realization of further control of the information epidemic, create more favorable conditions for the prevention and emergency management of the negative impact of the information epidemic, and contribute to the further advancement of global recovery.

### Research limitations and future research directions

From the perspective of cognition and emotion, this study investigated the influence of topic types, sentiment types, and emotion types on information dissemination, and further explored the conditional role of media factors and time factors in the process of information dissemination. However, some limitations should be acknowledged, and these limitations are the direction of our further research in the future. First, this study used the methods of Bert+Top2vec and BiLSTM to study the topic variables, sentiment variables, and emotion variables. However, the black box principle of deep learning methods still requires us further explore the methods and realize innovation, to further improve the accuracy and precision of tag prediction. For example, combined with existing studies, we can compare the adopted deep learning method with other most advanced methods to improve the accuracy and efficiency of method recognition and obtain more authoritative recognition effects (Li et al., 2020a, 2020b, 2020c, 2020d). At the same time, in future research, we will also consider a variety of methods, such as graph clustering to analyze the mechanism of information transmission in social networks. As a classical method of real complex network analysis, the graph clustering method can provide us with other important perspectives for analyzing information transmission (Li et al., 2022). Secondly, the data used in this study came from the Weibo platform. Although we controlled variables such as sentence complexity, content length, and the existence of URLs and hashtags to reduce the interference of confounding factors, the limitations of social media data make it difficult for us to obtain the individual attribute characteristics of users. This makes it more difficult for us to further reduce the interference of hybrid factors such as user attributes. Therefore, we will consider combining social media data with survey data in future work, making up for the deficiency of social media research through questionnaire surveys, enriching data content, and obtaining more comprehensive and in-depth results. Finally, considering the difficulty of data acquisition, the research only used the Weibo platform as our data acquisition platform. In future research, we will consider collecting relevant information about the same event from multiple platforms, to improve the applicable scope of the research results. At the same time, we also consider including different types of emergencies in the analysis and increasing the comparison of different emergencies. It can further enrich the research content and get more authoritative conclusions.

## Conclusions

Based on the theory of planned behavior, sentiment contagion theory, emotional evaluation theory, resource mobilization theory, and life cycle theory, this study took large-scale social media samples as data sources to carry out empirical research. Based on the cognitive dimension and sentiment dimension, this study used deep learning methods to generate topic variables, sentiment variables, and emotion variables and then investigated the influence of information attributes on information dissemination. Moreover, the study also introduced media factors and time factors to investigate the conditional role played by the number of followers, user types, and life cycles. The research results confirmed that there were differences in the dissemination of information with different topics, sentiments, and emotional types on social media, in which user influence and life cycles played a moderating role. The research results have contributed to the prediction and management of public opinion information and the maintenance of network order, and are conducive to reducing the group pressure of network users, controlling the spread of negative emotions in the network, and reducing the negative impact of network group incidents. Relevant findings can further realize the emergency management of public health emergencies, and then promote the establishment of a more harmonious network society.

## Data Availability

The datasets generated during and/or analyzed during the current study are available from the corresponding author on reasonable request.
